# Two large inversions seriously suppress recombination and are essential for key genotype fixation in cabbage (*Brassica oleracea* L. var. *capitata*)

**DOI:** 10.1093/hr/uhae030

**Published:** 2024-01-30

**Authors:** Bin Zhang, Yuankang Wu, Shoufan Li, Limei Yang, Mu Zhuang, Honghao Lv, Yong Wang, Jialei Ji, Xilin Hou, Fengqing Han, Yangyong Zhang

**Affiliations:** State Key Laboratory of Vegetable Biobreeding, Institute of Vegetables and Flowers, Chinese Academy of Agricultural Sciences, Beijing 100081, China; State Key Laboratory of Crop Genetics and Germplasm Enhancement, College of Horticulture, Nanjing Agricultural University, Nanjing 210095, China; State Key Laboratory of Vegetable Biobreeding, Institute of Vegetables and Flowers, Chinese Academy of Agricultural Sciences, Beijing 100081, China; State Key Laboratory of Crop Genetics and Germplasm Enhancement, College of Horticulture, Nanjing Agricultural University, Nanjing 210095, China; State Key Laboratory of Vegetable Biobreeding, Institute of Vegetables and Flowers, Chinese Academy of Agricultural Sciences, Beijing 100081, China; State Key Laboratory of Vegetable Biobreeding, Institute of Vegetables and Flowers, Chinese Academy of Agricultural Sciences, Beijing 100081, China; State Key Laboratory of Vegetable Biobreeding, Institute of Vegetables and Flowers, Chinese Academy of Agricultural Sciences, Beijing 100081, China; State Key Laboratory of Vegetable Biobreeding, Institute of Vegetables and Flowers, Chinese Academy of Agricultural Sciences, Beijing 100081, China; State Key Laboratory of Vegetable Biobreeding, Institute of Vegetables and Flowers, Chinese Academy of Agricultural Sciences, Beijing 100081, China; State Key Laboratory of Vegetable Biobreeding, Institute of Vegetables and Flowers, Chinese Academy of Agricultural Sciences, Beijing 100081, China; State Key Laboratory of Crop Genetics and Germplasm Enhancement, College of Horticulture, Nanjing Agricultural University, Nanjing 210095, China; State Key Laboratory of Vegetable Biobreeding, Institute of Vegetables and Flowers, Chinese Academy of Agricultural Sciences, Beijing 100081, China; State Key Laboratory of Vegetable Biobreeding, Institute of Vegetables and Flowers, Chinese Academy of Agricultural Sciences, Beijing 100081, China

## Abstract

Chromosomal inversion is an important structural variation that usually suppresses recombination and is critical for key genotype fixation. In a previous study, an 11.47 Mb recombination suppression region was identified in the yellow–green leaf locus *BoYgl-1* on chromosome 1, but the cause of recombination suppression is still unclear. In this study, chlorophyll and carotenoid contents were found to be significantly decreased in the yellow–green leaf mutant YL-1. Genome assembly and comparative analysis revealed that two large inversions in YL-1 were responsible for the severe recombination suppression in the *BoYgl-1* locus. Analyses with inversion-specific markers revealed that the inversions were present in 44 (including all wild cabbage; INV1 and INV2) of 195 cabbage inbred lines and 15 (INV1) ornamental kale inbred lines, indicating that these species with INV1 or INV2 may have evolved much earlier than other types of cabbage. Analyses with inversion-correlated markers revealed that the genotypes of CoINV1, CoINV2 and CoINV3 were highly correlated with INV1 and INV2, indicating that INVs could fix the key genotypes of the involved region. In addition, a 5.87 Mb assembly inversion was identified at the *BoYgl-1* locus in the TO1000 genome by genome comparative analysis. This study provides new insight into the recombination suppression mechanism of chromosomal inversion and the application of genome fragment fixation in cabbage breeding.

## Introduction

Chromosomal inversion is a classic type of structural variation produced by the reinsertion of segments bound by breakpoints in the reverse orientation that usually inhibits genetic recombination at the involved locus. In rice, the severe recombination suppression in a floral organ number locus *FON3* was caused by a paracentric inversion, which limits the fine mapping of *FON3* candidate genes [1]. In tomato, Seah et al. identified a 300 kb chromosomal inversion that severely suppresses genetic recombination in the nematode resistance locus *Mi-1* [2]. Rönspies et al. induced a 17.1 Mb chromosomal inversion by CRISPR–Cas-mediated chromosome engineering in *Arabidopsis*. A massive reduction in the recombination frequency was detected in the inverted chromosome region by SNP-based genotyping [3].

Chromosomal inversion plays an important role in fixing favorable allelic combinations by suppressing genetic recombination and is essential for maintaining the association with coadapted genotypes. Inversions are also important for creating a new open reading frame, changing the expression pattern of genes near the breakpoints, and analyzing species evolution [3, 4]. In peach, Zhou et al. reported that a 1.7-Mb chromosomal inversion located ~3 kb downstream of the *PpOFP1* gene is responsible for flat/round fruit shape. The transcriptional activation of *PpOFP1* by chromosomal inversion can repress the vertical elongation of fruit at the early developmental stage, resulting in a flat fruit shape [5]. In *Arabidopsis*, a paracentric 1.17-Mb inversion associated with fecundity under drought was identified on chromosome 4. SNP analysis revealed that the inversion originated from Southeastern Europe and the *FRI-Col* allele originated from Northwest Europe [6].

In addition to recombination suppression, assembly errors in a reference genome also affect the fine mapping of some key genes. Han et al. reported that Scaffold000063 was reverse assembled in the 02-12 reference genome when mapping the petal color gene *cpc-1* in cabbage [7]. Liu et al. reported that the fine-mapping region of the purple leaf gene *BoPr* in cabbage was located not in the primary-mapping region but on an unanchored scaffold Scaffold000035_P2, revealing possible assembly error in the 02-12 reference genome^8^. Cabbage is an important vegetable with leaf head as consumer product, and leaf color is a crucial commercial trait in cabbage. It is also important to identify the leaf color gene and reveal the molecular regulatory mechanism underlying leaf color formation in cabbage breeding.

In our previous study, Liu et al. identified an 11.47 Mb recombination suppression region (with a recombination rate of 13.65 Mb/cM) in the yellow–green leaf locus *BoYgl-1* on chromosome 1 [9]. In the present study, we performed genome assembly comparison and correlation marker analysis to reveal the cause of recombination suppression and the role of recombination suppression in key genotype fixation. This finding lays a foundation for revealing the action mechanism of chromosomal inversion and applying genetic exchange in cabbage breeding.

## Results

### Characterization of the YL-1 mutant

**Figure 1:**
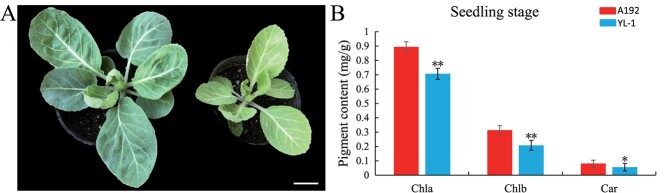
Phenotypes of the parental lines and quantification of pigments. **A** A192 with normal-green leaves (left) and YL-1 with yellow–green leaves (right). Bar = 3 cm. **B** Chl a, Chl b and carotenoid contents in the leaves of A192 and YL-1 at the seedling stage. Error bars represent the standard errors of three biological replicates (Student’s *t*-test: **P < 0.01).

Compared with A192 (normal–green leaf), the YL-1 mutant exhibited a stunted growth and yellow–green leaf phenotype throughout its entire life cycle (same growth time) (Fig. 1A). The yellow–green leaf phenotype is controlled by a single recessive gene according to genetic analysis [17]. Photosynthesis pigment content analysis revealed that the Chl a, Chl b and carotenoid contents were significantly reduced in YL-1 compared with A192 at the seedling and mature stages (Fig. 1B; Supplementary Fig. S1). These results suggest that *BoYgl-1* is involved in Chl biosynthesis in vivo.

### Two large inversions result in severe recombination suppression in the *BoYgl-1* locus

**Figure 2:**
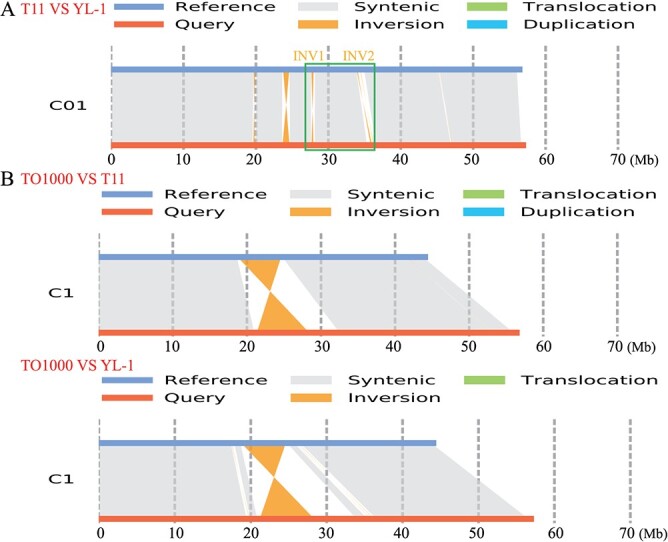
Analysis of recombination suppression in the *BoYgl-1* locus. A Genome C01 chromosome comparison between T11 and YL-1. The green box indicates the recombination suppression region with two inversions. B Genome C01 chromosome comparisons of YL-1 with TO1000 and T11 with TO1000.

In a previous study, an 11.47 Mb recombination suppression region (C01: 18,069,792-29,537,314 bp) was identified in the yellow–green leaf locus *BoYgl-1* on chromosome 1 based on the TO1000 genome [9]. To clarify the cause of recombination suppression in the *BoYgl-1* locus, the YL-1 genome was sequenced and assembled through a combination of three technologies (Illumina, PacBio and Hi-C), resulting in a 540.01 Mb assembly with a contig N50 size of 21.39 Mb (Supplementary Fig. S2 and Table S1). By comparing the YL-1 and T11 (a new assembly of TO1000) genomes, two large inversions (C01: 27,674,008-27,939,251 bp; C01: 35,834,373-36,026,024 bp) were identified near C01 30 Mb in YL-1. According to the TO1000 genome, inversion 1 corresponds to the region C01: 19,167,451-19,468,797 bp (301 kb), and inversion 2 corresponds to the region C01: 25,728,351-25,853,969 bp (126 kb). INV1 and INV2 were both located in the detected recombination suppression region (C01: 18,069,792-29,537,314 bp) (Fig. 2A), indicating that INV1 and INV2 may be responsible for the recombination suppression in the *BoYgl-1* locus. In addition, genome comparisons of YL-1 with TO1000 and T11 with TO1000 showed that the region of C01: 18,750,286-24,619,815 bp (5.87 Mb) in the *BoYgl-1* locus was inversely assembled in the TO1000 genome (Fig. 2B), which lays a foundation for the identification of *BoYgl-1* gene.

**Figure 3:**
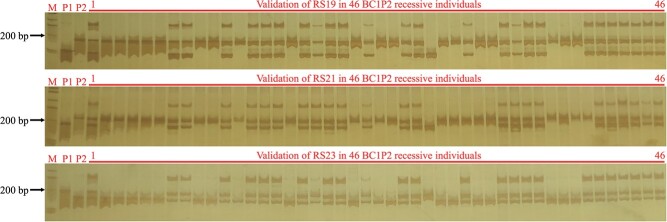
Amplicons of the InDel markers RS19, RS21 and RS23 in parents and 46 recessive individuals of the BC_1_P_2_ population. M represents the DNA ladder, P_1_ is the normal-green leaf inbred line A192, P_2_ is the yellow–green leaf mutant 522Y, and lanes 1–46 are 46 recessive individuals with yellow–green leaves.

**Figure 4:**
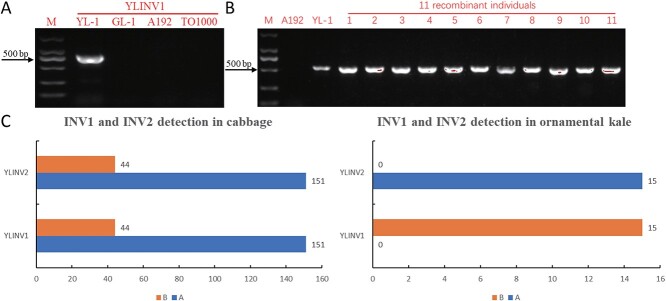
Verification of inversion-specific markers YLINV1 and YLINV2. A Amplicons of the marker YLINV1 in parents (YL-1, GL-1, 11-192 and TO1000). B Amplicons of the marker YLINV1 in 11 recessive individuals of the BC_4_P_2_-BC_6_P_2_ populations. M represents the DNA ladder, and lanes 1–11 represent 11 recessive individuals with yellow–green leaves. C Genotype statistics of the markers YLINV1 and YLINV2 in 195 different cabbage (including 4 wild cabbage) and 15 ornamental kale inbred lines (with normal-green leaves). A represents the genotype consistent with A192, and B represents the genotype consistent with YL-1.

Subsequently, to detect the recombination rate at this region in other mapping population, three polymorphic InDel markers with a size of approximately 200 bp (at the 19.80 Mb, 21.01 Mb and 23.39 Mb of C01; Supplementary Table S2) were developed by comparing the resequencing data of the parents A192 and another yellow–green leaf mutant 522Y (without INV1 and INV2) based on the TO1000 genome. Markers RS19, RS21 and RS23 were then used to determine whether recombination suppression was also present in this region by validation in 46 recessive individuals of the BC_1_P_2_ population. The results showed that the recombination rate in this region was 0.23 Mb/cM, which was higher than the normal level in the cabbage genome (~0.6 Mb/cM), strongly indicating that the two large inversions in YL-1 result in severe recombination suppression in the *BoYgl-1* locus (Fig. 3 and Fig. 4).

### Evolutionary analysis of INV1 and INV2

Using the inversion-specific markers YLINV1 and YLINV2, we first verified the parents (YL-1, GL-1, A192 and TO1000) and 11 recessive individuals (with yellow–green leaves) of the BC_4_P_2_-BC_6_P_2_ populations. The results showed that the target bands could be amplified only in YL-1 and 11 recessive individuals, which was consistent with expectations (Fig. 4A and B; Supplementary Fig. S3). Markers YLINV1 and YLINV2 were then used to determine whether inversion 1 and inversion 2 were present in 195 different cabbage (including 4 wild cabbage and 522Y mutant) and 15 ornamental kale inbred lines. The results revealed that INV1 and INV2 were both present in 44 of the 195 cabbage inbred lines, and these 44 inbred lines mainly consisted of overwintering cabbage, Brussels sprout and all wild cabbage (without 522Y). Only INV1 existed in all ornamental kale inbred lines (Fig. 4C). These markers exhibited high accuracy and can be used for efficient identification of INV1 and INV2 in cabbage. Overall, the analyses suggested that INV1 and INV2 of YL-1 may have originated from the wild cabbage and that these species with INV1 or INV2 may have evolved much earlier than other types of cabbage.

### INV1 and INV2 are essential for key genotype fixation

**Figure 5:**
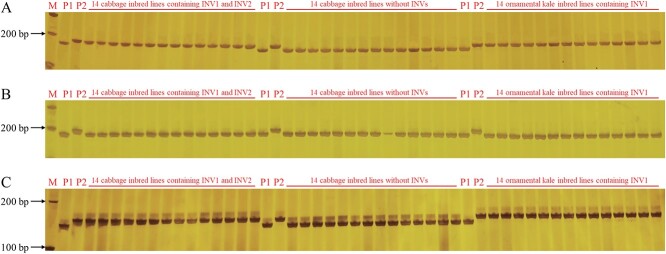
Correlation analysis between genotypes of CoINV1, CoINV3 and CoINV4 and INV1 and INV2. A Amplicons of the marker CoINV1 in 14 cabbage inbred lines containing INV1 and INV2, 14 cabbage inbred lines without INV1 and INV2, and 14 ornamental kale inbred lines containing only INV1. B Amplicons of the marker CoINV4 in 14 cabbage inbred lines containing INV1 and INV2, 14 cabbage inbred lines without INV1 and INV2, and 14 ornamental kale inbred lines containing only INV1. C Amplicons of the marker CoINV3 in 14 cabbage inbred lines containing INV1 and INV2, 14 cabbage inbred lines without INV1 and INV2, and 14 ornamental kale inbred lines containing only INV1. M represents the DNA ladder, P_1_ represents the A192, and P_2_ represents the YL-1.

According to the ‘TO1000’ reference genome, 18, 11 and 461 genes were identified in the INV1, INV2, and between INV1 and INV2 regions, respectively (Supplementary Table S3). By comparing the resequencing data between A192 and YL-1, only one variant gene was identified in INV1 and INV2, respectively, and markers CoINV1 (on *Bo1g063850*, encodes plastid ribosomal protein CL9) and CoINV2 (on *Bo1g087490*, encodes an ER-localized ABC transporter with a role in seed development) were developed accordingly. We also selected an intergenomic variation in INV1 and developed marker CoINV3. We then randomly selected three important variant genes and developed markers CoINV4 (on *Bo1g078560*, encodes plastid ribosomal protein S11), CoINV5 (on *Bo1g080190*, encodes CGR2 that plays an important role in plant growth) and CoINV6 (on *Bo1g083530*, encodes an F-box protein that is essential for plant growth) in the region between INV1 and INV2. Markers CoINVs were then used to detect the correlation between these variations and INVs in 14 cabbage inbred lines containing INV1 and INV2, 14 cabbage inbred lines without INV1 and INV2, and 14 ornamental kale inbred lines containing only INV1. The results showed that the genotypes of CoINV4, CoINV5 and CoINV6 in all inbred lines were consistent with A192, and the genotypes of CoINV1, CoINV2 and CoINV3 in all inbred lines containing INV1 or INV2 were consistent with YL-1. The genotypes of CoINV1, CoINV2 and CoINV3 were highly correlated with INV1 and INV2, indicating that INVs could fix the key genotypes of the involved region and suppress its genetic recombination in cabbage hybrid breeding (Fig. 5; Supplementary Fig. S4).

## Discussion

Chromosomal inversion usually results in severe recombination suppression. In rice, Jiang et al. identified a paracentric inversion at the *FON3* locus associated with floral organ number, which severely suppressed genetic recombination in this region [1]. In *Arabidopsis*, a 17.1 Mb chromosome inversion was induced by CRISPR/Cas9 technology, SNP-based genotyping showed that the recombination frequency in the inverted region was greatly reduced [3]. In this study, two large inversions (INV1 and INV2) were detected at the yellow–green leaf locus *BoYgl-1* in YL-1 by comparative genomic analysis, and INV1 and INV2 were found to be the cause of recombination suppression in the *BoYgl-1* locus by inversion-specific markers validation. This is also the first report of chromosome inversion in cabbage.

The nonhomologous end joining (NHEJ) pathway is an important mechanism underlying chromosomal inversion. In this study, 301-kb INV1 and 126-kb INV2 were probably induced by chromosomal DNA double-strand breaks along with illegitimate DNA end joining via the NHEJ pathway. INV1 and INV2 were both present in 44 (including overwintering cabbage, Brussels sprout and all wild cabbage) of the 195 cabbage inbred lines, and INV1 existed in all 15 ornamental kale inbred lines according to analyses with YL-1 inversion-specific markers. Our study gives rise to the hypothesis that these species containing INV1 or INV2 may have evolved much earlier than other types of cabbage. In addition, chromosomal inversions usually suppress the genetic recombination of sets of epistatically interacting genes. Genes involved in an inversion may segregate as a single Mendelian locus and may be selected during adaptation. In this study, the genotypes of CoINV1 and CoINV2 were completely correlated with INV1 and INV2, indicating that INVs could fix the key genotypes of the involved region and that cabbage species with INVs may show selective advantages under certain survival conditions [1, 5, 10, 11].

Pentatricopeptide repeat (PPR) proteins play essential roles in plant growth and development. According to their different motifs, PPR proteins can be classified into the P and PLS subfamilies. Most PPR proteins localize to the chloroplast or mitochondrion and participate in posttranscriptional RNA modification [12–14]. In rice, Chen et al. identified the yellow–green leaf mutant *pgl12*. PGL12 is a PLS-type PPR protein targeted to chloroplasts that is required for chloroplast development and 16S rRNA processing [15]. In *Arabidopsis*, Wang et al. identified the P-type PPR protein ECD2 localized to chloroplasts, and the *ecd2* mutant exhibited a cotyledon-specific albino phenotype [16]. Feng et al. identified a pale-green leaf 2 mutant (*Gmpgl2*) in soybean. GmPGL2 is a chloroplast-localized PLS-type PPR protein that is essential for C-to-U RNA editing via the formation of a complex RNA editosome [17]. In addition, Toda et al. identified a mitochondrion-localized PLS-type PPR protein MPR25 in rice, and the *mpr25* mutant showed a phenotype of pale-green leaves and growth retardation [18]. In maize, two PLS-DYW subfamily PPR proteins, EMP17 and PPR27, targeted to the mitochondria were identified, and homozygous kernels of both mutants were small and white, with an empty pericarp phenotype [19, 20]. In this study, a PPR gene, *Bo1g078420*, involved in mitochondrial RNA processing, was identified in the *BoYgl-1* locus. In rice, the *MPR25* (mitochondrial PPR-encoding) mutant shows a growth retardation and pale-green leaf phenotype highly similar to that of YL-1 [18]. Thus, we designated *Bo1g078420* as a strong candidate gene for *BoYgl-1*. *Bo1g078420* contains 13 PPR motifs and belongs to the P subfamily (based on the Pfam database: http://pfam.sanger.ac.uk./search?tab=searchSequenceBlock) (Supplementary Fig. S5B). Sequence analysis revealed that a 1-bp deletion, 24 SNPs in the promoter region and five SNPs (containing three nonsynonymous mutations) in the coding region of *Bo1g078420* were identified in YL-1 (Supplementary Fig. S5A and S6; Supplementary Table S2). The expression level of *Bo1g078420* in yellow–green leaf YL-1 was significantly decreased compared with normal-green leaf A192 (Supplementary Fig. S5C). Taken together, the sequence variations and the expression change in *Bo1g078420* may be responsible for the formation of yellow–green leaves in cabbage.

## Materials and methods

### Plant materials

The female parent A192 (P_1_) is a Chinese kale inbred line with normal green leaves; the male parent YL-1 (P_2_) is a cabbage inbred line with yellow–green leaves. A192 was crossed with YL-1 to produce F_1_ lines. BC_4_P_2_-BC_6_P_2_ populations were generated by successive backcrosses of F_1_ × YL-1. All plant materials used in this study were grown in a greenhouse (16 h light/8 h dark photoperiod; 25 °C ± 3 °C) at the Institute of Vegetables and Flowers, Chinese Academy of Agriculture Sciences (IVFCAAS, Beijing, China).

### Pigment content analysis

Chlorophyll and carotenoid contents were determined as previously described [21]. Fresh leaves (~0.2 g) of A192 and YL-1 were collected at the seedling stage, and then placed in 5 ml of 80% acetone for 24 h in the dark. Chl a, Chl b and carotenoid contents were measured using a DU800 spectrophotometer (Beckman Coulter, USA) at wavelengths of 663, 645 and 470 nm, respectively. Three biological replicates were performed per sample.

### Genome sequencing assembly and comparative analysis

High-quality genomic DNA was extracted from 4-week-old seedling fresh leaves of YL-1 using the FastPure Plant DNA Isolation Mini Kit (Vazyme, Nanjing, China). The genomic DNA was subsequently used for Illumina, PacBio and Hi-C library construction and sequencing. Illumina libraries with an insert size of 250 bp and PacBio libraries with an insert size of 25 kb were sequenced on an Illumina platform and the PacBio Sequel platform (Pacific Biosciences), respectively. The Hi-C libraries were sequenced on an Illumina Hi-Seq 4000 sequencer. Library construction, sequencing and genome assembly were carried out by Biomarker Technologies Co., Ltd. (Beijing, China). Genome comparisons of YL-1 with TO1000, T11 (a new assembly of TO1000) with TO1000, and YL-1 with T11 were performed as previously reported [22].

### Recombination suppression analysis

The recombination rate in a region was calculated by the ratio of physical and genetic distance between two markers. If the recombination rate in a region is four times lower than the normal recombination level in the cabbage genome (~600 kb/cM), we consider that recombination suppression is present in this region.

### Analysis of YL-1 inversion-specific markers

According to the YL-1 genome, YLINV1 (inversion-specific marker 1) with a size of 537 bp and YLINV2 (inversion-specific marker 2) with a size of 318 bp were designed (Supplementary Table S2) on the left boundary of inversion 1 (INV1, C01: 27,674,008 bp) and inversion 2 (INV2, C01: 35,834,373 bp), respectively. Markers YLINV1 and YLINV2 were then used to detect inversion 1 and inversion 2 in 11 recessive individuals of the BC_4_P_2_-BC_6_P_2_ populations, 195 different cabbage (including 4 wild cabbage) and 15 ornamental kale inbred lines.

### Analysis of YL-1 inversion-correlated markers

Based on the *B. oleracea* ‘TO1000’ reference genome (http://plants.ensembl.org/Brassica_oleracea/Info/Index) and the resequencing data of INV1, INV2, and between INV1 and INV2 regions of A192 and YL-1, six inversion-correlated markers CoINV1 (on the *Bo1g063850* gene in INV1, C01: 19,176,898 bp), CoINV2 (on the *Bo1g087490* gene in INV2, C01: 25,761,861 bp), CoINV3 (on the intergenic region in INV2, C01: 25,785,767 bp), CoINV4 (on the gene *Bo1g078560* between INV1 and INV2, C01: 23,050,171 bp), CoINV5 (on the gene *Bo1g080190* between INV1 and INV2, C01: 23,742,642 bp) and CoINV6 (on the gene *Bo1g083530* between INV1 and INV2, C01: 24,202,580 bp) with a size of approximately 200 bp were designed (Supplementary Table S2). Markers CoINVs were then used to detect the correlation between these variations and inversions in 14 cabbage inbred lines containing INV1 and INV2, 14 cabbage inbred lines without INV1 and INV2, and 14 ornamental kale inbred lines containing only INV1.

### Candidate gene analysis of *BoYgl-1*

Based on the annotations of the ‘TO1000’ reference genome, genes located within the candidate interval were analyzed to identify the yellow–green leaf gene *BoYgl-1*. The gene-specific marker GS420 (primers GS420-F and GS420-R) was used to amplify the promoter and genomic sequences of *Bo1g078420*. The sequencing and expression analysis (primers qRT420-F and qRT420-R) of the candidate gene were performed as described previously [23]. All primers used for candidate gene analysis are listed in Supplementary Table S2.

## Supplementary Material

Web_Material_uhae030

## Data Availability

All the data generated or analyzed in this study are included in this published article and its supplementary information files. All the sequence data of the present study have been deposited in the NCBI Sequence Read Archive (SRA) database under BioProject PRJNA949422. The reference genome ‘TO1000’ can be found from the website https://plants.ensembl.org/Brassica_oleracea/Info/Index. The genome assembly can be found through the NCBI Sequence Read Archive (SRA) database under BioProject PRJNA1060854.

## References

[1] Jiang L , Zhang W, Xia Z. et al. A paracentric inversion suppresses genetic recombination at the *FON3* locus with breakpoints corresponding to sequence gaps on rice chromosome 11L. Mol Gen Genomics. 2007;277:263–72 10.1007/s00438-006-0196-7 17143630

[2] Seah S , Yaghoobi J, Rossi M. et al. The nematode-resistance gene, *Mi-1*, is associated with an inverted chromosomal segment in susceptible compared to resistant tomato. Theor Appl Genet. 2004;108:1635–42 14963654 10.1007/s00122-004-1594-z

[3] Rönspies M , Schmidt C, Schindele P. et al. Massive crossover suppression by CRISPR–Cas-mediated plant chromosome engineering. Nat Plants. 2022;8:1153–9 36109610 10.1038/s41477-022-01238-3

[4] Crown KN , Miller DE, Sekelsky J. et al. Local inversion heterozygosity alters recombination throughout the genome. Curr Biol. 2018;28:2984–2990.e3 30174188 10.1016/j.cub.2018.07.004 PMC6156927

[5] Zhou H , Ma R, Gao L. et al. A 1.7-Mb chromosomal inversion downstream of a *PpOFP1* gene is responsible for flat fruit shape in peach. Plant Biotechnol J. 2021;19:192–205 32722872 10.1111/pbi.13455 PMC7769229

[6] Fransz P , Linc G, Lee CR. et al. Molecular, genetic and evolutionary analysis of a paracentric inversion in *Arabidopsis thaliana*. Plant J. 2016;88:159–78 27436134 10.1111/tpj.13262 PMC5113708

[7] Han F , Cui H, Zhang B. et al. Map-based cloning and characterization of *BoCCD4*, a gene responsible for white/yellow petal color in *B. oleracea*. BMC Genomics. 2019;20:242 30909886 10.1186/s12864-019-5596-2 PMC6434876

[8] Liu X , Gao B, Han F. et al. Genetics and fine mapping of a purple leaf gene, *BoPr*, in ornamental kale (*Brassica oleracea* L. var. *acephala*). BMC Genomics. 2017;18:230 28288583 10.1186/s12864-017-3613-x PMC5348804

[9] Liu X , Yu H, Han F. et al. Differentially expressed genes associated with the cabbage yellow-green-leaf mutant in the *ygl-1* mapping interval with recombination suppression. Int J Mol Sci. 2018;19:2936 30261688 10.3390/ijms19102936 PMC6212964

[10] Hoffmann AA , Sgrò CM, Weeks AR. Chromosomal inversion polymorphisms and adaptation. Trends Ecol Evol. 2004;19:482–8 16701311 10.1016/j.tree.2004.06.013

[11] Feuk L , Carson AR, Scherer SW. Structural variation in the human genome. Nat Rev Genet. 2006;7:85–97 16418744 10.1038/nrg1767

[12] Lurin C , Andrés C, Aubourg S. et al. Genome-wide analysis of Arabidopsis pentatricopeptide repeat proteins reveals their essential role in organelle biogenesis. Plant Cell. 2004;16:2089–103 15269332 10.1105/tpc.104.022236 PMC519200

[13] Colcombet J , Lopez-Obando M, Heurtevin L. et al. Systematic study of subcellular localization of *Arabidopsis* PPR proteins confirms a massive targeting to organelles. RNA Biol. 2013;10:1557–75 24037373 10.4161/rna.26128 PMC3858439

[14] Barkan A , Small I. Pentatricopeptide repeat proteins in plants. Annu Rev Plant Biol. 2014;65:415–42 24471833 10.1146/annurev-arplant-050213-040159

[15] Chen L , Huang L, Dai L. et al. *PALE-GREEN LEAF12* encodes a novel pentatricopeptide repeat protein required for chloroplast development and 16S rRNA processing in rice. Plant Cell Physiol. 2019;60:587–98 30508149 10.1093/pcp/pcy229

[16] Wang X , An Y, Qi Z. et al. PPR protein early chloroplast development 2 is essential for chloroplast development at the early stage of *Arabidopsis* development. Plant Sci. 2021;308:110908 34034865 10.1016/j.plantsci.2021.110908

[17] Feng X , Yang S, Zhang Y. et al. *GmPGL2*, encoding a pentatricopeptide repeat protein, is essential for chloroplast RNA editing and biogenesis in soybean. Front Plant Sci. 2021;12:690973 34567023 10.3389/fpls.2021.690973 PMC8458969

[18] Toda T , Fujii S, Noguchi K. et al. Rice *MPR25* encodes a pentatricopeptide repeat protein and is essential for RNA editing of *nad5* transcripts in mitochondria. Plant J. 2012;72:450–60 22747551 10.1111/j.1365-313X.2012.05091.x

[19] Liu R , Cao S, Sayyed A. et al. The DYW-subgroup pentatricopeptide repeat protein PPR27 interacts with ZmMORF1 to facilitate mitochondrial RNA editing and seed development in maize. J Exp Bot. 2020;71:5495–505 32531050 10.1093/jxb/eraa273

[20] Wang Y , Liu XY, Huang Z. et al. PPR-DYW protein EMP17 is required for mitochondrial RNA editing, complex III biogenesis, and seed development in maize. Front Plant Sci. 2021;12:693272 34394147 10.3389/fpls.2021.693272 PMC8357149

[21] Zhang T , Dong X, Yuan X. et al. Identification and characterization of *CsSRP43*, a major gene controlling leaf yellowing in cucumber. Hortic Res. 2022;9:uhac212 36479584 10.1093/hr/uhac212 PMC9719040

[22] Yang Z , Ge X, Li W. et al. Cotton D genome assemblies built with long-read data unveil mechanisms of centromere evolution and stress tolerance divergence. BMC Biol. 2021;19:115 34082735 10.1186/s12915-021-01041-0 PMC8176745

[23] Parkin IA , Koh C, Tang H. et al. Transcriptome and methylome profiling reveals relics of genome dominance in the mesopolyploid *Brassica oleracea*. Genome Biol. 2014;15:R77 24916971 10.1186/gb-2014-15-6-r77 PMC4097860

[24] Zhang B , Chen W, Li X. et al. Map-based cloning and promoter variation analysis of the lobed leaf gene *BoLMI1a* in ornamental kale (*Brassica oleracea* L. var. *acephala*). BMC Plant Biol. 2021;21:456 34615469 10.1186/s12870-021-03223-y PMC8496080

