# Supplementary material for: Two large inversions seriously suppress recombination and are essential for key genotype fixation in cabbage (Brassica oleracea L. var. capitata)
Source: Hortic Res. 2024 Jan 30;11(4):uhae030. doi: 10.1093/hr/uhae030 (PMC11784747; doi:10.1093/hr/uhae030)
Supplement: Web_Material_uhae030 [file web_material_uhae030.zip › Figure S4.pdf]

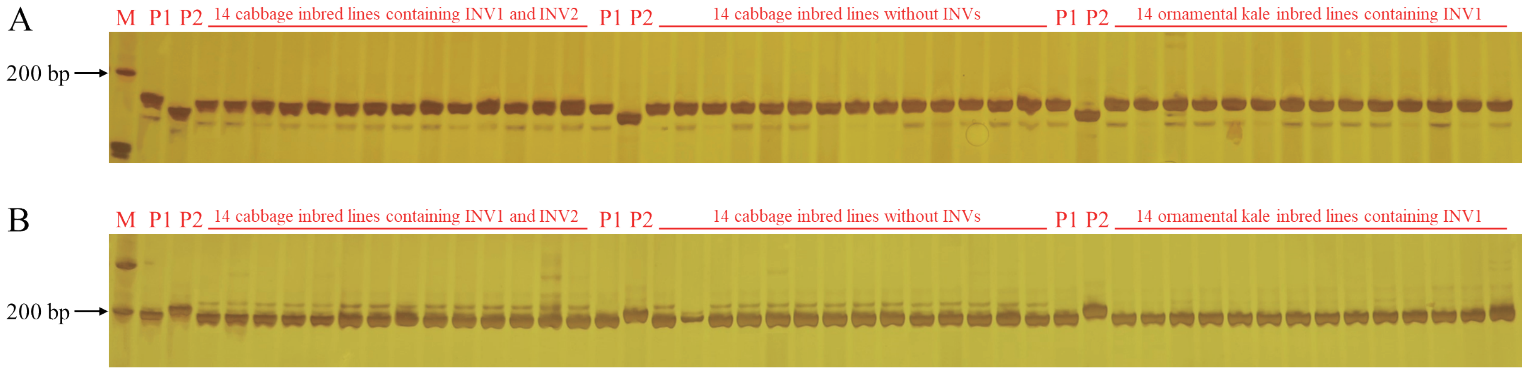

C

A192 CAGTTAAAAAAAAAACAACT
YL-1 CAGTT - AAAAAAAAACAACT
A1-A14 CAGTT - AAAAAAAAACAACT
B1-B14 CAGTTAAAAAAAAAACAACT
C1-C14 CAGCTAAAAAAAAAAAAACT
