# Supplementary material for: Two large inversions seriously suppress recombination and are essential for key genotype fixation in cabbage (Brassica oleracea L. var. capitata)
Source: Hortic Res. 2024 Jan 30;11(4):uhae030. doi: 10.1093/hr/uhae030 (PMC11784747; doi:10.1093/hr/uhae030)
Supplement: Web_Material_uhae030 [file web_material_uhae030.zip › Figure S6.pdf]

```
A192-ProBoYgl.seq   TTTCTCGCAGGTTGCCGAGCCCAAGATCTCCGGGGTGTTACTCTTGTTGAGTCATCTGCTTCGATG   66
YL-ProBoYgl.seq     TTTCTCGCAGGTTGCCGAGCCCAAGATCTCCGGGGTGTTACTCTTGTTGAGTCATCTGCTTCGATG   66

A192-ProBoYgl.seq   ATTATGGAGGTTATTCTTTTGACTCAATCTTTTCAATTTAAAGCGAATCTTTGGTCTTGTTCCTTC   132
YL-ProBoYgl.seq     ATTATGGAGGTTATTCTTTTGACTCAATCTTTTCAATTTAAAGCGAATCTTTGGTCTTGTTCCTTC   132

A192-ProBoYgl.seq   AGTTCAAGCTCGAAACTGCTCTCAGCTATCGATCTCTTCGTGAATGAGCTCAAGATAGAGATCTAA   198
YL-ProBoYgl.seq     AGTTCAAGCTCAAAACTGCTCTCAGCTATCGATCTCTTCGTGAATGAGCTCAAGATAGAGATCTAA   198

A192-ProBoYgl.seq   GTCTTTTCATGGAATGGGCCTACATCGTTGCTAGGCCGTGTCAAGATTTGAGTCTAACAATTGTCT   264
YL-ProBoYgl.seq     GTCTTTTCATGGAATGGGCCTACATCGTTGCTAGGCCGTGTCAAGATTTGAGTCTAACAATTGTCT   264

A192-ProBoYgl.seq   CCAAGTTCTTTAAGGATGATATCTCTTACAATTAATCTTTGAATAATTTTTCTCGCTGATATACGC   330
YL-ProBoYgl.seq     CCAAGTTCTTTAAGGATGATATCTCTTACAATTAATCTTTGAATAATTTTTCTCGCTGATATACGC   330

A192-ProBoYgl.seq   TTTTCAAGATTTTCCGTTGGTTTCGTGGGATATGATCCCCATCGTTTAAGTATAATTCTTCCTTAT   396
YL-ProBoYgl.seq     TCTTCAAGATTTTCCGTTGGTTTCGTGGGATATGATCCCCATCGTTTAAGTATAATTCTTCCTTAT   396

A192-ProBoYgl.seq   TGCAATCTTTTTGAGGCTATAAATCTCGCAACCACACCTTCCTTTGGTTGTACTTGTCTTCCTAGC   462
YL-ProBoYgl.seq     TGCAATCTTTTTGAGGCTATAAATCTCGCAACCACACCTTCCTTTGGTTGTACTTGTCTTCCTAGC   462

A192-ProBoYgl.seq   CCATCCCCATTTTTTTCAAAACGGGGTCTACCAGGAAGAAGCCCAGCTAGCGGGAATCTGAAAGGA   528
YL-ProBoYgl.seq     CCATCCCCATTTTTTTCAAAACGGGGTCTACCAGGAAGAAGCCCAGCTAGCGGGAATCTGAAAGGA   528

A192-ProBoYgl.seq   TTCACAGCATGATTGGTGTGGGTGGTTCTGAAAATATCCAGATGTGAGTGACTCTCTTACGGAAGT   594
YL-ProBoYgl.seq     TTCACAGCATGATTGGTGTGGGTGGTTCTGAAAAGATCCAGATGTGAGTGATTCTCTTACGGAAGT   594

A192-ProBoYgl.seq   ACTGAGAGAGGAAACACGTCTCCCACACTCAGTGGGTGGTGACACCAAGCCTAGCAGCGGTGATGT   660
YL-ProBoYgl.seq     ACTGAGAGAGGAAACACGTCTCCCACGCTCAGTTGGTGGTGACACCAAGCCTAGCAGAGGTGATGT   660

A192-ProBoYgl.seq   TGTTTCGGTGGGTGTCGTTCGTACGATTAGCCTGTTGGGTTCCGATGGTCGAGATCACAGACTAAA   726
YL-ProBoYgl.seq     TGTTTCGGTGGGTGTCGTTCGTACGATTAGCCTGTTGGTTTCCGATGGTCGAGATCACCGACTAAA   726

A192-ProBoYgl.seq   TAAGGCAAAAATTGCCAACATGGATTTGAGGTCGAGCTCGTCGAATGATGGTGCTGGCGTGAGACC   792
YL-ProBoYgl.seq     TAAGGCGAAAATTGCCAACATGGATTTGAGGTCGAGCTCGTCGAGTGATGGTGCTGGCGTGAGACC   792

A192-ProBoYgl.seq   TTTCCATTGGCGATTCTCTGAGGCGAAAGATTTCCCCATAACAAAAGATCCAGATAGCGTTGCTCA   858
YL-ProBoYgl.seq     TTTCCATTGGCGATTCTCTGAGGCGAAAGATTTCCCCATAACCAAAGATCCAGATAGCGTTGCTCA   858

A192-ProBoYgl.seq   TCTAGTAAGACACTTCAAGTATGCCGGTAGTCTGTTCATGTCTCTTCGGAATATGATGGAATGTGA   924
YL-ProBoYgl.seq     TCTAGTAAGACACTTCAAGTATGCCGCTAGTCTGTTCATGTCTCTTCGGAATATGATGGAATGTGA   924

A192-ProBoYgl.seq   CACATGTGTAAAGACGTTGGTTGCTCATGCCAAGGTGCCACCTTTGGATCTGATATCTGCTTTCAC   990
YL-ProBoYgl.seq     CACATGTGTAAAGACGTTGGTTGCTCATGCCAAGGTGCCACCTTTGGATCTTATATCTGCTTTCAC   990

A192-ProBoYgl.seq   TATTGTACTCATCTTCCTTTAAATGAGGTCGTTTTTCAAGCTATGGAGGCTTAGAATGAGTTTGGT   1056
YL-ProBoYgl.seq     TATTATACTCATCTTCCTTTAAATGAGGTCGTTTTTCAAGCTATGGAGGCTTAGAATGAGTTTGGT   1056

A192-ProBoYgl.seq   GCGACTTTGGAGCAAATGTTGAAGGATGTCAAGTGTGATGATGAGCTTAACAAAGTCAAGAAGGTC   1122
YL-ProBoYgl.seq     GCGACTTTGGAGCAAATGTTGAAGGATGTCAAGTGTGATGATGAGCTTAACAAAGTCAAGAAGGTC   1122

A192-ProBoYgl.seq   GTAAGGGAGTTGAAAATCAGTTTGAATCTAACTCATGATCGAGAATGTGCCAACTCTGGCCAATTG   1188
YL-ProBoYgl.seq     GTAAGGGAGTTGAAAATCAGTTTGAATCTAACTCATGATCGAGAATGTGTCAACTCTGGCCAATTG   1188

A192-ProBoYgl.seq   GCTGATGCCTAGAAGCTTGGGAATCAAGTTGTTTCTCTCGAAGCTCGACTCCGGATGATCTGGAAT   1254
YL-ProBoYgl.seq     GCTGATGCCTAGAAGCTTGGGAATCAAGTTGTTTCTCTCGAAGCTCGACTCCGGATGATCTGGAAT   1254

A192-ProBoYgl.seq   GAAAGAAAAGCAGCCCTCGAGAGGGTTTCTTTGCTGGAGGCGCAGATAGACAATTCGTCAGCTAAA   1320
YL-ProBoYgl.seq     GAAAGAAAATCAGCCCTCGAGAGGGTTTCTTTGCTGGAGGCGCAGATAGAGAATTCGTCAGCTAAA   1320

A192-ProBoYgl.seq   CATGTGGAGGATCTGCGTTTGGCTTCTTAGGAAGCTAATAAGATCCTTGCTGATAGCTACCTCGAC   1386
YL-ProBoYgl.seq     CATGTGGAGGATCTGCGTTTGGCTTCTTACGAAGCTAATAAGATCCTTGCTGATAGCTACCTCGAC   1386

A192-ProBoYgl.seq   GTGCTCTAAATGAGAAATGGGAGAGAAAGTAGGTTGCGACTGACTGTAAAGCTTGTCTTCGGAAGG   1452
YL-ProBoYgl.seq     GTGCTCTAAATGAGAAATGGGAGAGAAAGTAGGTTGCGACTGACTGTAAGGCTTGTCTTCGGAAGG   1452

A192-ProBoYgl.seq   TCATAGCTAACATCAATCTTATCGAAAAAAACTAGAGAATTTTTAAAAAGATGTTTGTGTAAAAAT   1518
YL-ProBoYgl.seq     TCATAGCTAACATCAATCTTATCGAAAAAAACTAGAGAATTTTTAAAAAGATGTTTGTGTAAAAAT   1518

A192-ProBoYgl.seq   ACAAAGACATAAATCTGAGAAAACTTATAAATTTACAATAGTTAGATTTGGAAATTACTAAACTTA   1584
YL-ProBoYgl.seq     ACAAAGACATAAATCTGAGAAAACTTATAAATTTACAATAGTTAGATTTGGAAATTACTAAACTTA   1584

A192-ProBoYgl.seq   CAAATATACAAATTGTATATATAAATTTCAGATCAAGTAAGTTACATAGTTTTTTAAACACCATTT   1650
YL-ProBoYgl.seq     CAAATTTACAAATTGTATATATAAATTTCAGATCAAGTAAGTTACATAGTTTTTTAAACACCATTT   1650

A192-ProBoYgl.seq   TAATTAGGTATGTTTTTGTAACTATGTTCATGCACCAATTGGTTAGTGTGCCATTGTACATCATCT   1716
YL-ProBoYgl.seq     TAATTAGGTATGTTTTTGTAACTATGTTCATGCACCAAT.AGTTAGTGTGCCATTGTACATCATCT   1715

A192-ProBoYgl.seq   ATTATTTTTCCAACAATAAATAGATATTTTGTAAAGTTAGTAGAAGTTGAGGGGTATATTCATCAA   1782
YL-ProBoYgl.seq     ATTATTTTTCCAACAATAAATAGATATTTTGTAAAGTTAGTAGAAGTTGAGGGGTATATTCATCAA   1781

A192-ProBoYgl.seq   TACCTTCAACTTTCTCACTACCTATCGAATTCCTGTTTTTTTTATTTTTTGTAAGAAAACAAAATC   1848
YL-ProBoYgl.seq     TACCTTCAACTTTCTCACTACCTATCGAATTCCTGTTTTTTTTATTTTTTGTAAGAAAACAAAATC   1847

A192-ProBoYgl.seq   CGAGCAGAATGAGAGAAAATGGCAAATACTGAGCAAAACCCTCGCTCAAAACGCCACCGTTTGAAT   1914
YL-ProBoYgl.seq     CGAGCAGAATGTGAGAAAATGGCAAATACTGAGCAAAACCCTCGTTCAAAACGCCACCGTTTGAAT   1913

A192-ProBoYgl.seq   CGCTTCCTCCGGCCAATCTAATCGTCGTCTCCACTCTCCAGCAGCTCAATCTATCGTCTCCGTCTG   1980
YL-ProBoYgl.seq     CGCTTCCTCCGGCCAATCTAATCGTCGTCTCCACTCTCCAGCAGCTCAATCTATCGTCTCCGTCTG   1979

A192-ProBoYgl.seq   TAAATTTAGAAGAAATAAGAAGACTCTCAAGTATTCATTGCC                           2022
YL-ProBoYgl.seq     TAAATTTAGAAGAAATAAGAAGACTCTCAAGTATTCATTGCC                           2021
```
