# Supplementary material for: Two large inversions seriously suppress recombination and are essential for key genotype fixation in cabbage (Brassica oleracea L. var. capitata)
Source: Hortic Res. 2024 Jan 30;11(4):uhae030. doi: 10.1093/hr/uhae030 (PMC11784747; doi:10.1093/hr/uhae030)
Supplement: Web_Material_uhae030 [file web_material_uhae030.zip › Supplementary information.pdf]

Supplementary information

Figure S1. Chl a, Chl b and carotenoid contents in the leaves of A192 and YL-1 at the mature stage. Error bars represent the standard errors of three biological replicates (Student's *t*-test: $^{**}P < 0.01$).

Figure S2. Chromatin contact heatmap of Hi-C data of YL-1.

Figure S3. Amplicons of the marker YLINV2 in parents and 11 recessive individuals of the BC_4P_2-BC_6P_2 populations. M represents the DNA ladder, and lanes 1–11 are 11 recessive individuals with yellow–green leaves.

Figure S4. Correlation analysis between genotypes of CoINV2, CoINV5 and CoINV6 and INV1 and INV2. A Amplicons of the marker CoINV5 in 14 cabbage inbred lines containing INV1 and INV2, 14 cabbage inbred lines without INV1 and INV2, and 14 ornamental kale inbred lines containing only INV1. B Amplicons of the marker CoINV6 in 14 cabbage inbred lines containing INV1 and INV2, 14 cabbage inbred lines without INV1 and INV2, and 14 ornamental kale inbred lines containing only INV1. M represents the DNA ladder, P_1 represents the A192 and P_2 represents the YL-1. C Sequencing results of the marker CoINV2. A1-A14 represent the 14 cabbage inbred lines containing INV1 and INV2, B1-B14 represent the 14 cabbage inbred lines without INV1 and INV2, and C1-C14 represent the 14 ornamental kale inbred lines containing only INV1.

Figure S5. Gene and protein structures and expression analysis of the *BoYgl-1*. A Gene structure of *Bo1g078420* with 5 SNPs in the coding region between A192 and YL-1. B Schematic diagram of the *Bo1g078420* protein with 13 PPR motifs. C Expression pattern of *Bo1g078420* as determined by qRT–PCR between A192 and YL-1. *BoActin* served as the equal loading control. Error bars represent the standard errors of three biological replicates (Student's *t*-test: $^{**}P < 0.01$).

Figure S6. Promoter sequence alignment of *BoYgl-1* between A192 and YL-1.

Table S1. Assembly statistics of the YL-1 genome.

Table S2. Primer sequences of the markers used in this study.

Table S3. The 490 predicted genes in the INV1, INV2, and between INV1 and INV2 regions.
