# Supplementary figures and images for: Two large inversions seriously suppress recombination and are essential for key genotype fixation in cabbage (Brassica oleracea L. var. capitata)
Source: Hortic Res. 2024 Jan 30;11(4):uhae030. doi: 10.1093/hr/uhae030 (PMC11784747; doi:10.1093/hr/uhae030)

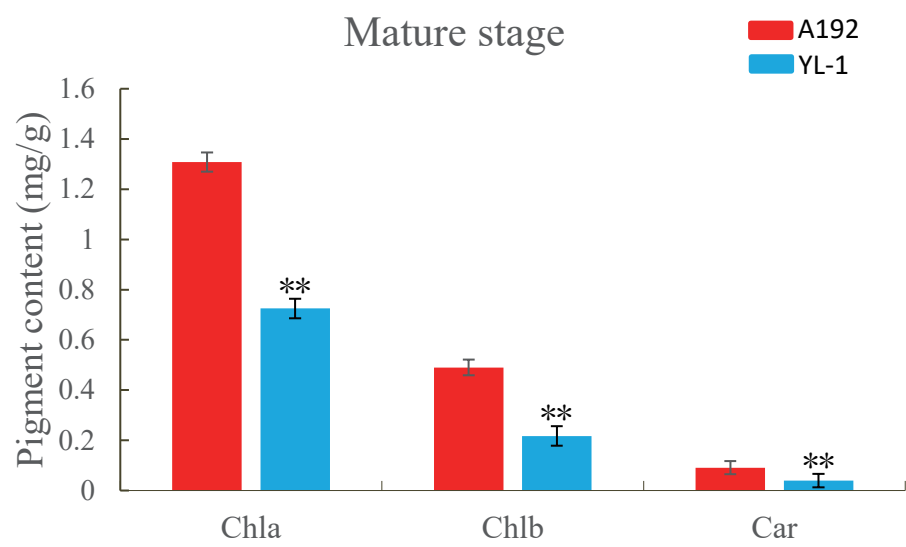

Supplement: Web_Material_uhae030 [file web_material_uhae030.zip › Figure S1.pdf]

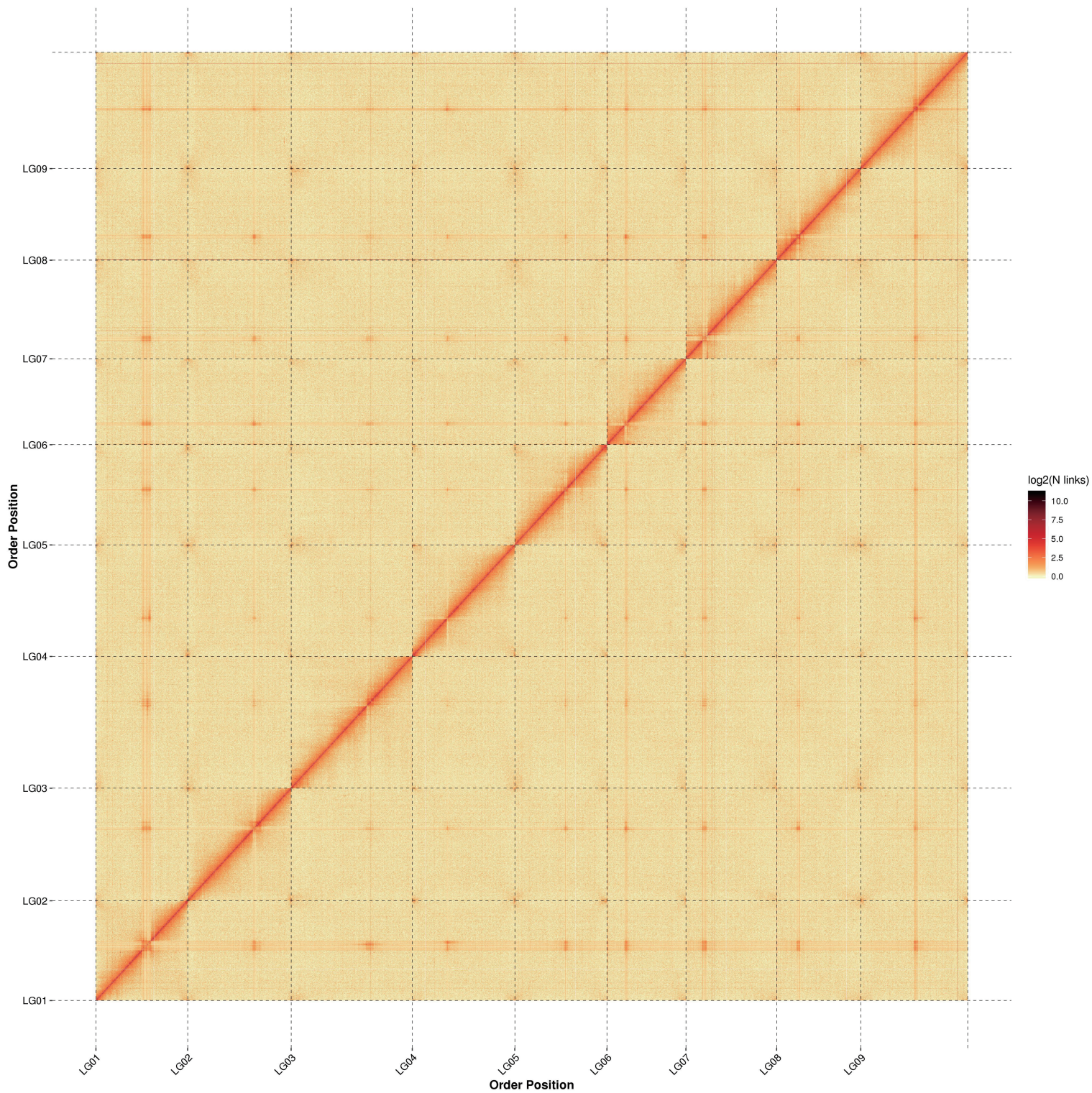

Supplement: Web_Material_uhae030 [file web_material_uhae030.zip › Figure S2.pdf]

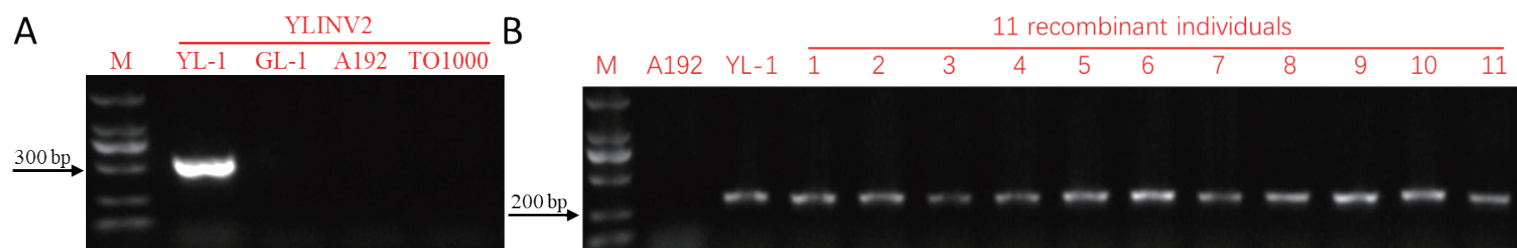

Supplement: Web_Material_uhae030 [file web_material_uhae030.zip › Figure S3.pdf]

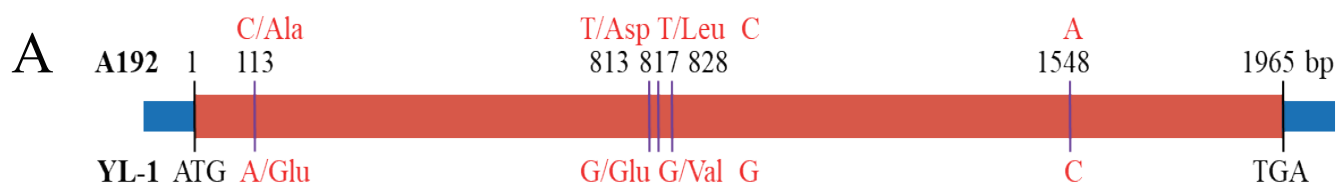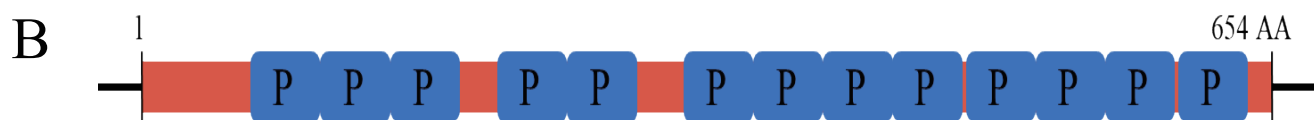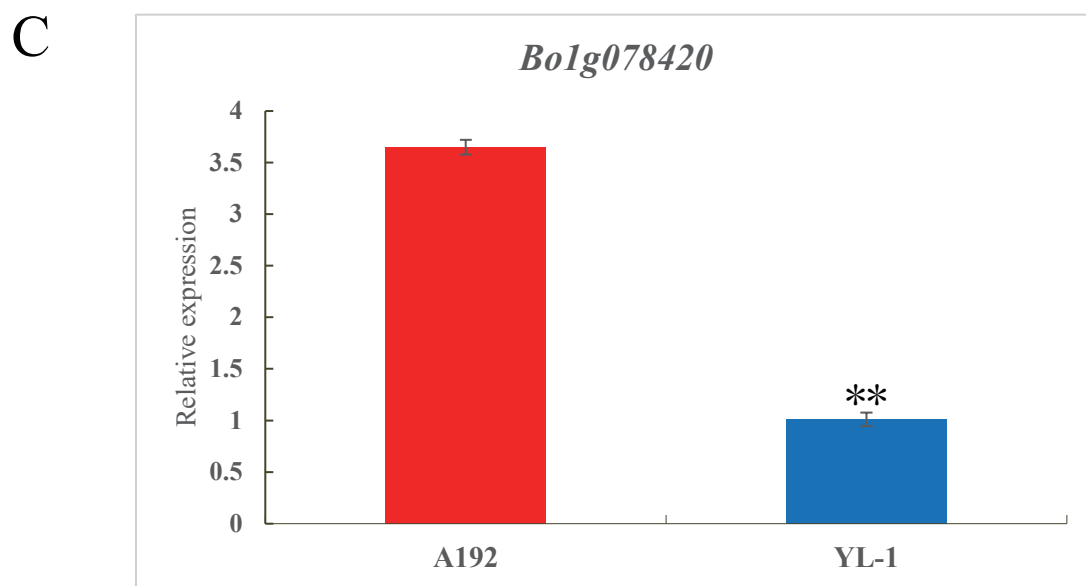

Supplement: Web_Material_uhae030 [file web_material_uhae030.zip › Figure S5.pdf]
